# 

**Published:** 1892-07

**Authors:** 


### CONTENTS:

ORIGINAL ARTICLES: PAGE

The Etiology of Southern Cattle Plague—Texas Fever. By Frank S. Billings... 397
Enlarged Mesenteric Gland and an Aneurism of the Anterior Mesenteric Artery. By J. E. Miller, M. R. C. V. S. ... 425
Notes on Parasites. By M. Francis... 426
A Peculiar Case of Ligation of the Small Intestines By C. A. Cary, B. S., D. V. M. 427
Review of the Avian Tuberculosis. By S. E. Weber, V. S... 429
Locoed Horses. By Frank A. McCullaugh... 435
United States Veterinary Medical Association... 437
New York State Veterinary Medical Society... 438

SELECTION:

Crib-biting... 439

NECROLOGY:

Death of Henri F. Formad... 447

SOCIETY PROCEEDINGS:

Keystone Veterinary Medical Association... 449
The Philadelphia Society of Veterinary Medicine... 450

BOOKS AND PAMPHLETS RECEIVED... 452

